# Preparation and Characterization of the Composition of Volatile Compounds, Fatty Acids and Thermal Behavior of Paprika

**DOI:** 10.3390/foods12102041

**Published:** 2023-05-18

**Authors:** Thomas Dippong, Lacrimioara Senila, Laura Elena Muresan

**Affiliations:** 1 Department of Chemistry and Biology, Technical University of Cluj-Napoca, 76 Victoriei Str., 430122 Baia Mare, Romania; 2 INCDO-INOE 2000, Research Institute for Analytical Instrumentation, 67 Donath Street, 400293 Cluj-Napoca, Romania; lacri.senila@icia.ro; 3 Raluca Ripan’ Institute for Research in Chemistry, Babes Bolyai University, Fantanele, 30, 400294 Cluj-Napoca, Romania; laura_muresan2003@yahoo.com

**Keywords:** paprika, thermal behavior, VOCs, sensorial profile, fatty acids

## Abstract

This study aimed to investigate the thermal behavior and composition of volatile compounds, fatty acids and polyphenols in paprika obtained from peppers of different countries. The thermal analysis revealed various transformations in the paprika composition, namely drying, water loss and decomposition of volatile compounds, fatty acids, amino acids, cellulose, hemicellulose and lignin. The main fatty acids found in all paprika oils were linoleic (20.3–64.8%), palmitic (10.6–16.0%) and oleic acid (10.4–18.1%). A notable amount of omega-3 was found in spicy paprika powder varieties. The volatile compounds were classified into six odor classes (citrus (29%), woody (28%), green (18%), fruity (11%), gasoline (10%) and floral (4%)). The total polyphenol content ranged between 5.11 and 10.9 g GA/kg.

## 1. Introduction

Paprika is widely used within the food industry as a natural colorant (i.e., in soups, sausages, cheeses and snacks) due to its ability to improve upon the flavor of food through its characteristic taste and pungency [1]. Paprika is obtained by the dehydration of some pepper fruit varieties (*Capsicum annuum* L.) followed by milling of the dried pepper to obtain a fine powder [1]. Drying conditions affect the rehydration capacity of dehydrated paprika quite significantly [2]. The intensity of its characteristic red color is the main quality criterion of paprika powder, although this parameter depends on the variety of pepper used as well as the employed preparation method [1]. Most manufacturers, however, lack the knowledge to produce a safe and standardized food product to prevent contamination with any foreign matter, molds or toxins [3]. In paprika, the most important compounds are carotenoids and capsaicinoids, as well as vitamins E and C [2]. Carotenoids are responsible for the color of paprika, their content within the product being connected to the variety and ripeness of peppers alongside their growing condition (i.e., cool and rainy seasons tend to yield fruit with more β-carotene and technological factors) [2]. The types of pigments found confer paprika its particular color (yellow, green or red). The red pigments are specific to Capsicum species and reveal the presence of pungent capsaicinoids capsaicin and dihydrocapsaicin (dominant constituents), nordihydrocapsaicin and homocapsaicin (minor constituents) [2]. In addition, paprika spice has advantageous health properties, such as analgesic, anti-obesity, cardio-protective or neurologic properties, among others. Therefore, this spice is readily used in the pharmaceutical and cosmetic industries [4]. Recently, the adulteration of condiment powders, such as paprika, pepper, curry, chili and saffron, has increased. In the case of paprika, this nutritional integrity is important as it contains carotenoid pigments, neutral lipids such as tocopherols and vegetable oil [5].

The formation of volatile compounds that generate the characteristic aroma is caused by chemical conversions, such as hydrolytic reactions, amino acid conversion, oxidative degradation reactions of lipids (fatty acids) and carotenoids (lipid-soluble pigments), Maillard reactions and caramelization browning [6]. Some studies highlight the fact that the quality and quantity of aroma and flavor compounds of paprika are decisive parameters for quality control [1]. More than 125 volatile compounds have been identified in fresh and processed paprika, although the significance of these compounds for the aroma is not yet well known [1]. The main VOCs in paprika are esters and terpenoids, followed by other minor compounds, such as lipoxygenase derivatives, nitrogen and sulfur compounds, phenol derivatives, norcarotenoids, carbonyls, alcohols and other hydrocarbons [7]. Esters usually confer a fruity aroma, while many aldehydes, referred to as green leaf volatiles, can create a grassy aroma [7].

Paprika oil contains saturated (SFAs), monounsaturated (MUFAs) and polyunsaturated (PUFAs) fatty acids. SFAs are undesirable in large quantities because they can cause cardiovascular problems. Moreover, they are stable at room temperature, and the unsaturated fatty acids start to increase the fluidity and oxidation process, leading to the formation of free radicals. Replacing SFAs with PUFAs has positive benefits for cholesterol in the blood. Among all PUFAs, linoleic (C18:2) and α-linolenic acids (C18:3) are essential fatty acids for food [8]. Abbeddou and co-workers studied the fatty acid profile of paprika oleoresin. The following content was reported: linoleic acid C18:2 (55.97%), C16:0 (15.16%), C18:1 (13.81%), C18:1 (13.18%), C18:3 (5.11) and small quantities (below 2%) of C12:0, C14:0, C16:1, C18:0, C20:0, C22:0, C24:0 and C20:1 [9]. Zaki et al. (2013) analyzed red varieties of paprika and found that the fat present in paprika is in an esterified form with carotenoids [4]. The analyzed paprika presents a content of lipids of around 8%, a content of carbohydrates of approximately 55% and is rich in some metals (K, P, Ca, Mg) while being poor in Na. Linoleic (C18:2), oleic (C18:1) and palmitic (C16:0) acids are the most predominant FAs [4]. Paprika contains high quantities of PUFAs which have valuable properties in decreasing cholesterol levels and reducing the risk of obesity. Kim et al. (2017) investigated the use of red paprika on the lipid metabolism of obese male mice for eight weeks [10]. The results revealed that red paprika reduces obesity, fatty acid oxidation and lipid droplet size [10]. The fatty acid content depends on the geographical origin, meteorological conditions and production process. Paprika cultivation under traditional conditions was preferred because of its higher quality.

The methods used for oil extraction from plants are solvent extraction, maceration, cold-press extraction, steam distillation, CO_2_ extraction, ultrasound, enfleurage and microwave irradiation. Oil is the fluid separated from the plant in the presence of a solvent. The most common solvent used for the extraction of oil is n-hexane. Studies show that polar solvents can extract all fatty acids and are preferred for this purpose. In addition, extraction methods influence the amounts of fatty acids. Combining solvent extraction, microwave irradiation, ultrasonic extraction and supercritical carbon dioxide techniques could significantly improve oil performance [11]. Kostrzewa et al. (2022) used supercritical CO_2_ extraction of dry paprika and n-hexane, and the results showed that there is no difference between the two extraction methods. [11]. Ultrasound-assisted extraction is a simple and ecologically safe method for the extraction of oil. The ultrasonic extraction method increases the yield of active compounds such as antioxidants, phenols and fatty acids. The advantages of UAE were lower extraction time, lower volume of solvent and lower temperature of extraction, as well as higher efficiency [12].

This study focused on the determination of the key volatile compounds, the comparative analysis of fatty acids, polyphenol and thermal behavior among wild-harvested and commercial paprika samples obtained from pepper varieties from different countries. Ultrasound-assisted extraction (UAE) was applied to extract oil from paprika powder varieties. The interest of this study consists in the following: (i) the literature is enriched with the obtained extensive characterization of different paprikas, (ii) the relationship between the thermal behavior and the content of volatile compounds, cellulose, hemicellulose, lignin and fatty acids, (iii) the aroma profile and classification of volatile compounds by classes of chemical compounds and types of aromas and (iv) the statistical analysis of the content of volatile substances and fatty acids of the eight varieties of paprika.

## 2. Materials and Methods

### 2.1. Chemicals

The chemicals, including methanol (CH_3_OH), chloroform (CHCl_3_), potassium chloride (KCl), sodium sulfate (Na_2_SO_4_), isooctane (C_8_H_18_), potassium hydroxide (KOH), sodium hydrogen sulphate monohydrate (NaHSO_4_·H_2_O), ethanol (C_2_H_6_O), diethyl ether (C_2_H_5_)_2_O, phenolphthalein (C_20_H_14_O_4_), sodium chlorite (NaClO_2_), sulphuric acid (H_2_SO_4_) and sodium hydroxide (NaOH), all of analytical grade, were purchased from Merck (Darmstadt, Germany). The standard FAME mixture (Supelco 37 component FAME mix, CRM47885) was purchased from Sigma-Aldrich (St. Louis, MO, USA). Ultrapure water (18.2 MΩ·cm^−1^ at 20 °C) was obtained from a Direct-Q3 UV Water Purification System (Millipore, Molsheim, France).

### 2.2. Sample Description

Paprika 1 (P1) was obtained from red Kapia peppers of Romanian origin, light reddish-brown in color, very pleasant in smell, sweet feeling with a well-defined pepper aroma.

Paprika 2 (P2) was obtained from hot peppers, with Romania as the country of origin. The uniform powder had a specific smell of pepper with a spicy taste.

Paprika 3 (P3) was obtained from golden pepper, with Romania as the country of origin—a yellow-brown powder with a specific and pleasant smell.

Paprika 4 (P4) was obtained from hot peppers, with Morocco as the country of origin—light red-orange powder, with a specific smell of peppers and a sweet spicy taste.

Paprika 5 (P5) was obtained from red Kapia peppers, with Turkey as the country of origin, and it was characterized by a light red-orange color, specific sweet pepper smell and a weaker taste compared to the others.

Paprika 6 (P6) is a ground chili originating from India, with a light red-brown color and a very spicy, aromatic and pleasant taste.

Paprika 7 (P7) is obtained from red Kapia peppers originating from China. The taste and smell of this red-orange colored paprika powder is pleasant and specific but weakly pronounced compared to the rest of the samples.

Paprika 8 (P8) is obtained from Kapia peppers originating from Hungary. It is characterized by a red-brick color, with a taste and smell specific to this particular pepper assortment.

#### Paprika Preparation Methods

Each variety of paprika (P1–P8) was prepared under the same working conditions. After washing the peppers, they were cut into rings and placed in the oven at a temperature of 80 °C and then left to dry until they reached a light red-brown color and acquired a crunchy texture. After cooling, they were chopped.

All the samples were freeze-dried (FreeZone 2.5 LiterBenchtop freeze dry system, Labconco, Kansas, MO, USA) at −40 °C and −25 psi for 24 h to uniformize their moisture content. The freeze-dried samples were ground using an agate mortar and pestle to obtain homogenized powders. The moisture of the samples was determined by drying the samples to a constant mass at 105 °C in a universal oven (UFE 400, Memmert, Schwabach, Germany).

### 2.3. Extraction of Lipids from Paprika Powder

The dried samples (1 g) were extracted with 20 mL chloroform: methanol (2:1, *v*/*v*) and introduced into an ultrasonic bath (ISOLAB, Germany, tank dimensions: 150 × 138 × 65 mm^3^, tank volume 1.3 L, ultrasonic power: 60 W, frequency: 40 kHz) for 15 min (repeated for four times) at room temperature, according to Pohndorf et al. (2016) with modifications [13]. The obtained mixture was extracted with 10 mL KCl (0.74%). The extracts were centrifuged (10 min at 5000 rpm), and the organic phase was recovered. Finally, the organic phase was filtered using Na_2_SO_4_ to obtain a clear solution. The solvent was evaporated using the rotary evaporator Laborota 4010 (Heidolph, Schwabach, Germany), and the oil obtained was dried at 60 °C in an oven. The yield and the lipid content were calculated using Equation (1).

(1) $$\mathrm{Lipid}\;\mathrm{content}\;\left(\%\right)=\frac{m_{L}}{m_{P}}\;\times 100$$

where *m_L_* is the extracted lipid weight, and *m_p_* is the mass of dried paprika powder. 

#### 2.3.1. Lipid Extraction Yield 

The oils obtained using ultrasound extraction with chloroform: methanol (2:1, *v*/*v*) were converted to FAME using transesterification methods according to [14]. The fatty acids were separated on Zb-WAX, a polyethylene glycol column stationary phase suitable for separating fatty acids.

#### 2.3.2. Fatty Acid Methyl Esters (FAMEs)

Fatty acid compositions were determined using a gas chromatography coupled with flame ionization detector technique, after lipid extraction and transesterification to fatty acid methyl esters. The obtained lipids were converted into FAMEs by transesterification with potassium hydroxide. The samples (0.06 g) were dissolved in isooctane, treated with 0.2 mL methanolic potassium hydroxide solution (CH_5_KO_2_) (2 mol/L) and vigorously stirred for 30 s. Lastly, the mixture was treated with 1 g of sodium hydrogen sulphate to avoid saponification of methyl esters and neutralize excess alkali. Each oil sample was trimethylated and analyzed in three replicates. 

#### 2.3.3. Free Fatty Acid (FFA) Content from Extracted Oil

The free fatty acids were determined by titrating the oil obtained with KOH (0.1 M in ethanol). An amount of *m* g of oil was dissolved in a solvent mixture of ethanol: diethyl ether (1:1, *v*/*v*), using phenolphthalein (2%) as the indicator. The FFA content was calculated in accordance with Equation (2):

(2) $$\mathrm{FFA}=\frac{56.1*V*C}{m}\mathrm{mg}\;\mathrm{KOH}/g$$

where 56.1 is the molecular weight of KOH, *V* is the volume of KOH used for titration (mL), *C* is the concentration of KOH used for titration, and *m* is the biomass of oil used for analysis. 

#### 2.3.4. GC Analysis

The FAME content was determined using GC-FID (Agilent Technologies, Santa Clara, CA, USA, 6890 N) equipped with a Zebron ZB-WAX capillary column (30 m × 0.25 mm × 0.25 µm) and a flame ionization detector (FID, Agilent Technologies 7683). The gas carrier was helium with a constant flow rate of 1 mL min^−1^. The injection volume was 1 µL in a 1:20 split mode. The GC oven temperature program consists of three stages: 60 °C for 1 min, 60 to 200 °C (rate 10 °C min^−1^, 2 min), 200 to 220 °C (5 °C min^−1^, 20 min). The temperature of the injector and detector was set to 250 °C. FAs in samples were identified by comparing their retention time with that of the Supelco FAME standard mixture.

### 2.4. Thermal Analysis

The thermal behavior of all samples was evaluated based on thermal analysis (TG-DTA) carried out with a Mettler-Toledo TGA/SDTA851. The measurements were performed at a heating rate of 20 °C/min in a controlled atmosphere using air or nitrogen with a flow rate of 60 mL/min.

### 2.5. Volatile Composition

For the HS-SPME GC-MS analysis of volatile organic compounds, 3 g of ground paprika P1–P8 was transferred to a 20 mL headspace vial, and 3 mL of NaCl saturated solution was added to enhance the volatile organic compounds in the headspace and to inhibit any enzymatic reactions. The method was developed according to Martín et al., with improvement [1]. The headspace vials were sealed with crimp-top caps with TFE-silicone headspace septa (Thermo Fischer Scientific, Waltham, MA, USA). Each vial was incubated for 20 min at 60 °C. Afterward, the SPME fiber Divinylbenene/Carboxen/Polydimethylsiloxane (50 µm DVB/30 µm CAR/30 µm PDMS) was exposed for 15 min (60 °C) at the headspace of the sample to perform the HS-SPME extraction of volatile organic compounds. Furthermore, the extracted volatile organic compounds were desorbed for 7 min from the fiber coating into the Thermo Fischer Scientific Trace 1310 GC gas chromatograph injection port set at 250 °C. The volatile organic compounds were separated using a DB-WAX capillary column (30 m × 0.25 mm i.d. × 0.25 µm film thickness, J&W Scientific Inc. (Folsom, CA, USA)). Ultrahigh purity helium was used as a carrier gas at a linear velocity of 1 mL/min. The oven temperature program was as follows: initial temperature of 35 °C, heated to 180 °C at a rate of 5 °C·min^−1^, increased to 230 °C at a rate of 15 °C·min^−1^ and then held at a plateau for 7 min. Mass spectra were recorded in electron impact (EI) ionization mode at 70 eV using a TSQ 9000 MS, Thermo Fischer Scientific mass spectrometer. The quadrupole mass detector, ion source and transfer line temperatures were set at 150, 230 and 280 °C, respectively. Mass spectra were scanned in the range *m*/*z* 50–450 amu. VOCs were identified by comparing the mass spectra with the NIST 14 database system library and linear retention index. The criteria for compound identification required a mass spectrum matching score of ≥80%. The results were expressed as a percentage of the relative peak area (% RPA) of a peak in each paprika sample that was calculated by dividing the peak area by the total peak area of all identified peaks in each chromatogram. The total ion chromatogram (TIC) of each sample was used for peak area integration.

All measurements were conducted in triplicate, and data are presented as the mean ± standard deviation.

### 2.6. Antioxidant Characterization

Polyphenols were measured using the Folin–Ciocalteu colorimetric method using a Perkin Elmer Lambda 25 spectrophotometer to measure the blue complex at 760 nm, and gallic acid was used as a reference standard [15]. All measurements were conducted in triplicate, and data are presented as the mean ± standard deviation.

### 2.7. Cellulose, Hemicellulose and Lignin Content

The content of cellulose, hemicelluloses and lignin in paprika varieties was determined according to Senila et al. [16]. The content of holocellulose (cellulose and hemicelluloses) was determined by delignification of samples with NaClO_2_ in acetic acid (10%). The content of cellulose was determined by treatment of holocellulose with NaOH (17.5%). The lignin was determined as present residue after treatment of samples with 72% H_2_SO_4_ solution.

### 2.8. Statistical Analysis

For the statistical processing of the data, OriginPro Data Analysis and Graphing Software (OriginLab Corporation, Northampton, MA, USA) was used. Descriptive data analyses, including standard deviation and Pearson correlation, explained by very strong correlation (0.9–1), strong correlation (0.70–0.89), moderate correlation (0.40–0.7), weak correlation (0.10–0.39) and negligible correlation, were realized. Two different sets of variables, including fatty acids and volatiles and polyphenols, were evaluated in order to separate the geographical provenance and types of the paprika samples. The paprika samples were grouped according to sets of variable contents by Agglomerative Hierarchical Clustering (AHC) using the squared Euclidian distance and the Ward method for combining clusters, using XLStat software version 2019.3.2 (Addinsoft, Paris, France).

## 3. Results and Discussion

### 3.1. Thermal Behavior

**Figure 1:**
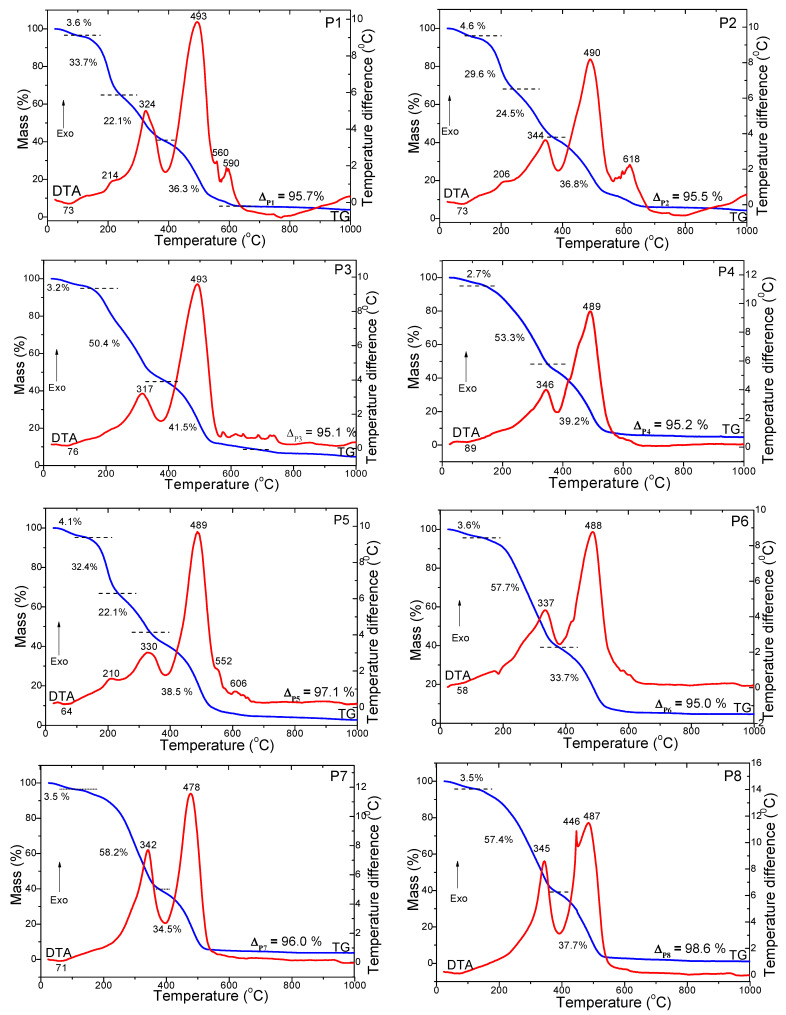
TG (blue line) and DTA (red line) curves of the paprika sample (P1–P8) under air atmosphere.

**Figure 2:**
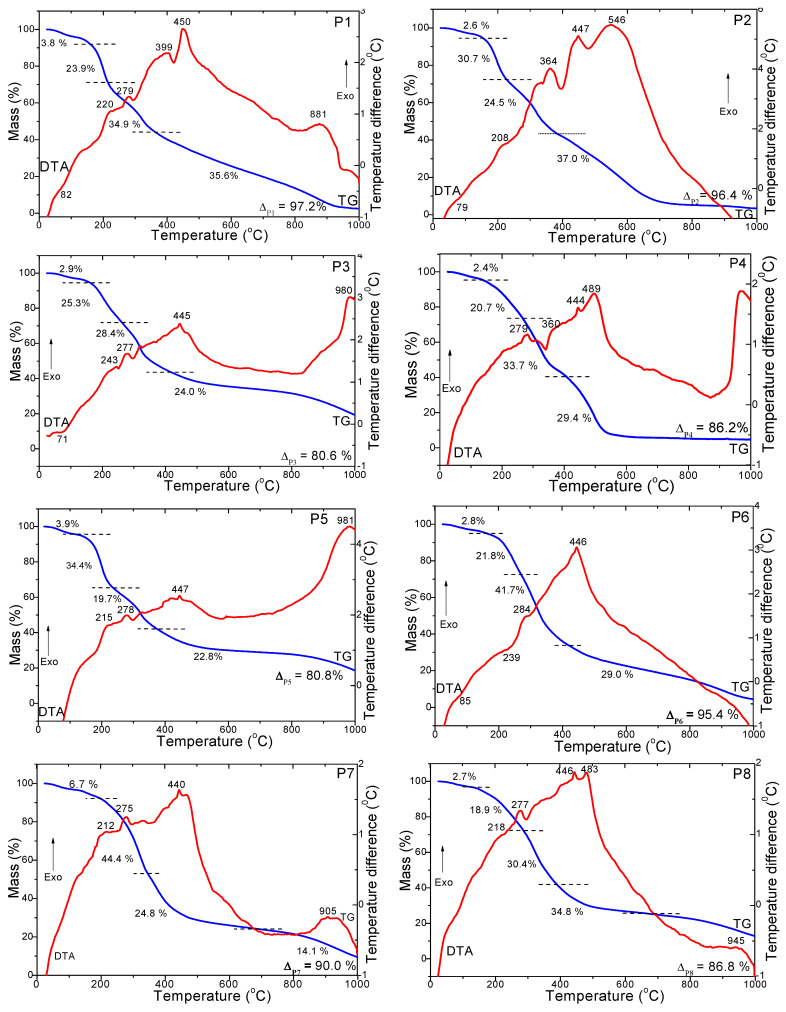
TG (blue line) and DTA (red line) curves of the paprika sample (P1–P8) under nitrogen atmosphere.

The decomposition stages of the paprika samples were investigated in both air (Figure 1) and nitrogen atmospheres (Figure 2) up to 1000 °C. All paprika samples have a similar thermal behavior in three or four stages under the air atmosphere. In the case of P1, P2 and P5, the DTA curve shows the first stage was characterized by an endothermic effect at 64–73 °C, accompanied by a mass loss of 3.6–4.6%, attributed to the evaporation of adsorbed water and solvent [17,18,19]. The second stage involved the decomposition of volatile compounds and polyphenols characterized by an exothermic effect at 206–214 °C, accompanied by a mass loss of 29.6–33.7% [17,18,19]. The third stage at 324–344 °C, with a mass loss of 22.1–24.5%, can be attributed to the decomposition of fatty acids and proteins [17,18,19]. The fourth stage of decomposition for P1 and P5 corresponds to the degradation of cellulose, hemicellulose and lignin, with three exothermic effects at 493–489 °C, 552–560 °C and 590–606 °C accompanied by a mass loss of 36.3%. In the case of P2, it was observed only through two exothermic effects at 490 °C (decomposition of hemicellulose and cellulose) and 618 °C (decomposition lignin), accompanied by a mass loss of 28.7 and 8.1% [14,15,16]. In the case of the P3, P4, P6, P7 and P8 samples, the DTA curve shows, for the first stage, an endothermic effect between 58 and 89% °C associated with a 2.7–3.6% mass loss on the TG curve. This can be attributed to the drying of paprika powders and the desorption of physically absorbed water molecules. For the second stage, an exothermic effect in the range of 317–346 °C associated with a mass loss of 50.4–58.2% on the TG curve is attributed to the decomposition of volatile compounds and polyphenols, fatty acids and proteins, and for third stage, an exothermic effect at 478–493 °C is associated with a mass loss of 33.7–41.5% corresponding to the degradation of cellulose, hemicellulose and lignin (only in the case of the P8 sample for the third stage a split peak in two exothermic effects at 446 and 487 °C can be observed) [17,18,19].

The thermal behavior is different for decompositions in a nitrogen atmosphere (Figure 2) compared to an air atmosphere (Figure 1). In all cases, five stages of decomposition can be discerned. The first stage of decomposition is observed by the endothermic effect on the DTA curve, with a mass loss of 2.4–6.7% corresponding to the evaporation of adsorbed water and solvent [17,18,19]. The second stage of visible decomposition through the exothermic effect from 210 to 220 °C, with a mass loss of 20.7–34.4%, can be attributed to the decomposition of volatile compounds and polyphenols [17,18,19]. The third stage of decomposition visible through the exothermic effect at around 243–284 °C, with a mass loss of 19.7–41.7%, is attributed to the decomposition of fatty acids, amino acids and proteins [17,18,19]. The fourth stage of decomposition, equivalent to a mass loss of 22.8–37.0%, was observable through exothermic effects at around 364–399 °C, 440–450 °C and 483–546 °C and can be attributed to the degradation of cellulose, hemicellulose and lignin [17,18,19]. In addition, for samples P1, P3, P4, P5, P7 and P8, an exothermic effect occurs at 881–981 °C, with a mass loss of around 14%, which occurs only in the nitrogen atmosphere. We infer that it could be attributed to mineral substances with nitrogen content present in the residue. The total mass losses were in the range of 95.0–98.6% in the air atmosphere compared to 80.6–97.2% in the nitrogen atmosphere. The thermal degradation stages of paprika varieties are in agreement with their compositions.

In our previous studies on coffee [20], the thermal analysis revealed various transformations in coffee composition, namely, drying, water loss and decomposition of polysaccharides, lipids, amino acids and proteins. Thermal analysis also revealed transformations in cocoa powder’s composition [21]: drying and water loss; decomposition of pectic polysaccharides, lipids, amino acids and proteins; and crystalline phase transformations and carbonizations.

### 3.2. Cellulose, Hemicellulose and Lignin Content of Paprika Samples

Lignin is a natural phenolic polymer found in higher plant tissues and the second most abundant polymer after organic cellulose [22]. In all samples, cellulose, hemicellulose and lignin contents were identified. The lignin content varied in the following order (%): P1 (15.2 ± 1.1) > P4 (13.1 ± 1.0) > P8 (13.0 ± 0.89) > P7 (12.8 ± 0.85) > P2 (11.3 ± 1.1) > P5 (11.2 ± 1.0) > P6 (8.3 ± 0.62) > P3 (7.2 ± 0.52). A high concentration of lignin was identified in red Kapia pepper. Our results are in accordance with other researchers’ results regarding lignin determination from pepper [23]. Estrada et al. [23] reported 4–9% lignin from the fruit of *Capsicum annum* pepper species and reported the variation of lignin during the maturation period. It was concluded that the maturation process caused the lignin content to decrease. The process can be explained by the rearrangement of cell structure by plant maturation and the interaction between lignin-like substances derived from phenyl propanoid precursors [23]. Celluloses and hemicelluloses are carbohydrates that provide the taste of the pepper and can create links with protein, lipids and biomolecules in the paprika powder [24].

The cellulose content varied in the following order (%): P2 (32.2 ± 2.1) > P4 (28.6 ± 1.8) > P6 (25.5 ± 2.0) > P1 (23.1 ± 1.9) > P5 (23.0 ± 1.3) > P7 (21.1 ± 1.6) > P8 (19.2 ± 1.2) > P3 (19.1 ± 1.0), whereas hemicellulose contents were (%) P6 (13.5 ± 0.8) > P4 (12.1 ± 1.0) > P2 (11.1 ± 0.9) > P8 (8.9 ± 0.5) > P1 (8.1 ± 0.5) > P5 (7.2 ± 0.6) > P7 (5.4 ± 0.3) > P3 (5.2 ± 0.4). The highest content of cellulose was found in spicy varieties. According to Mudrić et al., in Serbian paprika, the following sugars were identified: glucose, fructose, arabinose, xylose, mannose, disaccharides (trehalose and maltose), trisaccharides and sugar alcohols [24]. The results show a good agreement between the thermogravimetric analysis and the presence of cellulose, hemicelluloses and lignin in paprika samples.

### 3.3. HS-SPME GC-MS Analysis of Volatile Organic Compounds

Volatile compounds of Romanian paprika were analyzed using SPME followed by GC-MS, as shown in Table 1. This study identified 32 volatile compounds divided into seven classes in the samples, including 16 hydrocarbons, 5 aldehydes, 4 ketones, 3 alcohols, 2 esters, 1 sulfur compound and 1 heterocyclic compound (Table 1).

**Figure 3:**
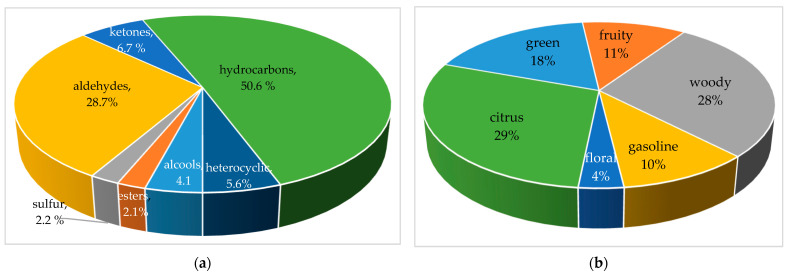
Classification of volatile compounds identified in paprika by chemical class (**a**) and aroma profile (**b**).

Hydrocarbons had the highest proportion (50.6%, Figure 3a) in the samples, inducing fruity, camphorous, sweet, lemony, pine-like, minty, woody, resinous, balsamic, plastic, roasted, citrus, floral, aromatic, green, chemical, herbaceous, clove, pepper or spicy odors [6,25]. The limonene was the most abundant in the paprika samples, although its amount varied between samples as follows: P7 (100%) > P8 (42.8%) > P6 (41.6%) > P3 (30.2%). M-cymene may be formed from oxidation of monoterpene hydrocarbons by isomerization and oxidation [26,27].

Aldehydes (28.7%, Figure 3a) are formed through the oxidative degradation of amino acids during their interaction with sugars at high temperatures or the interaction of amino acids and polyphenols in the presence of polyphenol oxidase and mainly contribute to fatty and cowy flavor [6,7,25]. The formation of aldehydes indicates that the Maillard reaction occurred during the heating or roasting processes, correlated to the concentrations of free amino acids present in the samples [3,28]. The aromas given by aldehydes are fruity, fatty, green, oil, green grassy, very strong, harsh and lemon-like [6]. Furfural (highest content in P1, P2, P3 and P5 paprika samples) was the major volatile component in this group and is usually an indicator of thermal damage during roasting [3,29]. Hexanal (P4, P6 and P8), as an oxidation product of enzymatic as well as autoxidative linoleic acid oxidation, increased strongly with heating, and is the main compound found in fresh pepper and is responsible for the odor of freshly cut grass or ground leaves of green plant materials [1,3,28,30]. Benzaldehyde, the only odor-active aromatic aldehyde, only present in the B2 sample, is characterized by a caramel-like or roasted odor and commonly exists as the glycosidically bound form in paprika [6,27].

The content of ketones (6.7%, Figure 3a) in paprika samples is associated with fruity, spicy, cinnamon, banana, mushroom, camphor, cedar leaf, mint and bitter aromas [6,15]. Oxidative decomposition of unsaturated fatty acids is the main pathway for ketone formation during heating treatments. In addition, oxidative decomposition of saturated fatty acids could produce volatiles, such as alkenes and alcohols, which might be further oxidized to produce ketones under a high temperature treatment [7,27,31]. 2-Heptanone (only present in sample P2) contributed to ‘stale’ and ‘cabbage’ odors and very little to the entire aroma formation because of its high threshold and low content [6]. Carvone (only in samples P3, P5 and P6) provided ‘mint’, ‘basil’ and ‘fennel’ odors and was detected as one of the odor-active compounds of paprika [6].

Alcohol compounds (4.1%, Figure 3a) account for cooling, camphoraceous, fresh pine, ozone, citrus, floral, green, peppermint, woody, earth and sweet odors [6]. 2-Methyl-butanal (P1 and P2 samples) showed the highest abundance, which provided a characteristic roasted garlic odor [3].

Esters (2.1%, Figure 3a) in paprika samples induced fruity (apple, cherry, pear, etc.), floral, herbaceous, sweet, refreshing, green, grassy, bergamot, lavender and minty odors [6]. Esters were produced by oxidation or pyrolysis of unsaturated fatty acids in peppers under high temperature, especially pericarp and seeds, which could explain the increase in esters during the initial drying process [3,7,32].

Sulfur compounds are decomposition products of thiosulfinates and are derived from amino acid flavor precursors of the *Allium* family, including garlic and shallot [3,33,34]. Dimethyl disulfide (2.2% only in the P5 sample) (pungent, spicy) is a sulfur-containing volatile from garlic that is responsible for medicinal properties, presents the most abundant flavor in garlic oil and plays a major role in the formation of di- and trisulfides found as components of garlic [3,35].

The predominant aroma is citrus (29%), where the limonene and woody aroma (28%) of paprika is represented by furfural, 5-methyl furfural, α-pinene, β-pinene, 3-(bromomethyl)cyclohexane, benzaldehyde, β-myrcene, 1.3.8-p-menthathiene and cedrene [6]. Green-aroma-associated compounds (18%, Figure 3b), after the processing of peppers, showed that terpenes, sesquiterpenes and terpene derivatives are more abundant in pungent paprikas than sweet ones and 2-methylbutan-1-ol, dimethyl disulfide, hexanal, camphene, cis-myrtenol, terpinyl acetate, 2-n-pentylthiophene and phenylsulfanylacetaldehyde decreased upon maturation [6,36]. Fruity aroma (11%, Figure 3b) is represented by compounds such as 2-methylisovalerate, 2-heptanone, 2-Propanone, 4-carene, 2-methyl-7-norbornanol, β-phellandrene, β-ocimene, m-cymene, β-ocimene, γ–terpinene and 2-nonen-4-one. These compounds are sensitive to compositional alterations and variations in the metabolic pathways during ripening, harvest, post-harvest and storage and many factors related to the variety and type of technological treatment [6,36]. The gasoline flavor (10%) is given by 2-methyloctane, and the floral aroma (4%) is given by terpinolene and d-carvone [6].

### 3.4. Fatty Acid Content in Paprika Oil Varieties

**Figure 4:**
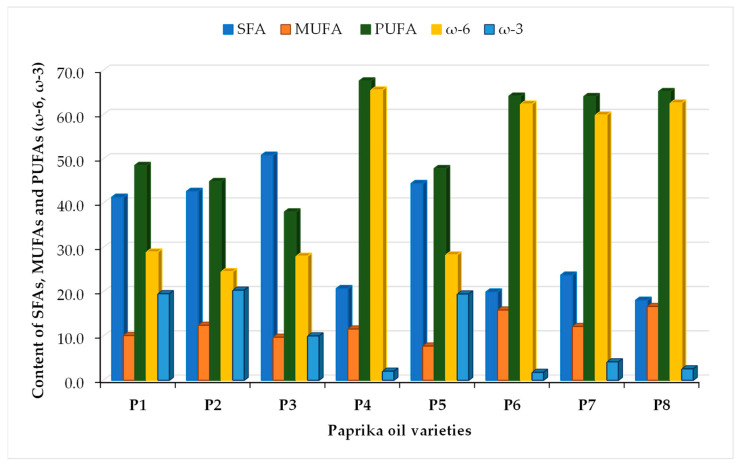
The content of SFAs, MUFAs and PUFAs.

However, the cis (oleic acid) and trans (elaidic acid) isomers of C18:1 and cis (linoleic acid) and trans (linolaidic acid) isomers of C18:2 were not separated and were quantified together. The lipid content varied in the following order: P5 (16.0 ± 1.1%) > P2 (15.5 ± 1.0%) > P6 (13.7 ± 1.1%) > P7 (13.0 ± 1.0%) > P8 (12.4 ± 0.89%) > P4 (12.1 ± 0.90) > P1 (10.01 ± 0.90%) > P3 (4.2 ± 0.2%). The fatty acid methyl esters found in all oil from paprika powder varieties are presented in Table 2 and Figure 4. All oil samples analyzed contain SFAs, MUFAs and PUFAs in different quantities within sample types. The SFAs found in paprika oil are C12:0, C14:0, C15:0, C16:0, C17:0, C18:0, C20:0, C21:0, C22:0 and C23:0. Palmitic acid (C16:0) is the predominant SFA in all paprika samples. The highest content of C16:0 was found in the P1 sample. The identified MUFAs are C14:1, C16:1, C17:1, C18:1(c + t) and C20:1. The highest oleic and octadecenoic acid (C18:1(c + t)) content was found in P8 (11.31%) and P4 (10.38%).

The PUFAs are divided into omega-6 (ω-6) and omega-3 (ω-3). The highest PUFA (ω-6) quantities found in all samples are reported for linoleic acid (C18:2(c + t)) and varied in the following order: P4 (64.7%) > P8(61.9%) > P6(61.4%) > P7 (56.8%) > P3(24.3%) > P1, P5 and P2 (approx.20%). The highest PUFA content was found in spicy samples. According to Figure 4, the highest content of SFA was found in golden pepper (P3), due to a high content of tricosanoic acid (C23:0). Red paprika varieties were found to be free of saturated tricosanoic acid. Rutkowska and Stolyhwo (2009) reported a content of 59.4% C18:2 and 5.1% C18:3 in red paprika powder oil. The method by which the oil was extracted was Soxhlet with hexane/ethyl ether (1:1) [37]. Several studies reported that food rich in PUFAs reduces the risk of cardiovascular diseases, and they were recently shown to prevent the risk of SARS-CoV-2 infection [38]. Omega-3 was found in high quantities in samples P2 (20.6%), P1 and P3 (19.5%) and in lower amounts in samples P7, P4, P8 and P6 (lower than 4%). The conclusion is that spicy paprika has a high omega-3 content and is appropriate for consumption. It is essential that the consumption of SFAs is replaced with the consumption of PUFAs. It is recommended that the consumption of SFAs should not exceed 10%, according to the Food and Agricultural Organization of United Nations [39]. A lower ω-6/ω-3 ratio is desirable due to the reduction in the risk of cardiovascular problems. The free fatty acids were analyzed in all samples (as acid values). All the oil samples contained below 1%, demonstrating the solubility of fatty acids in used solvents in the presence of the ultrasound method. The fatty acid profile of paprika varieties is important for food chemistry due to differences in variety chemistry, assigned quality, origin, food taste and color.

### 3.5. Total Polyphenol Contents

Polyphenols are often responsible for the antioxidant capacity of plant products, and they could be important constituents to explain the protective effects of plant-derived foods and beverages [2]. The results of the total polyphenolic content of paprika spices, measured with Folin–Ciocalteau reagent using the spectrometric method, are given in Table 3. Polyphenols are linked to health-promoting properties as they show antioxidant, anti-inflammatory, antidiabetic and anticarcinogenic activity [40]. The antioxidant character was attributed to the higher number of polyphenols present in the paprika and to the presence of lignin. The highest content of polyphenols was measured in P3 (10.9 g GA kg^−1^), and the lowest content was measured in P8 (5.11 g gallic acid/kg). The sample with the lowest total polyphenol content (sweet paprika spice) had only about 50% of the content of paprika delicate with the highest value; there is only a weak connection between the pungency of the spices and the polyphenolic amount [2]. The amount of polyphenols in paprika products could be influenced by the variety of pepper and could also be dependent on the time of harvesting and processing [2].

### 3.6. Principal Component Analysis

**Figure 5:**
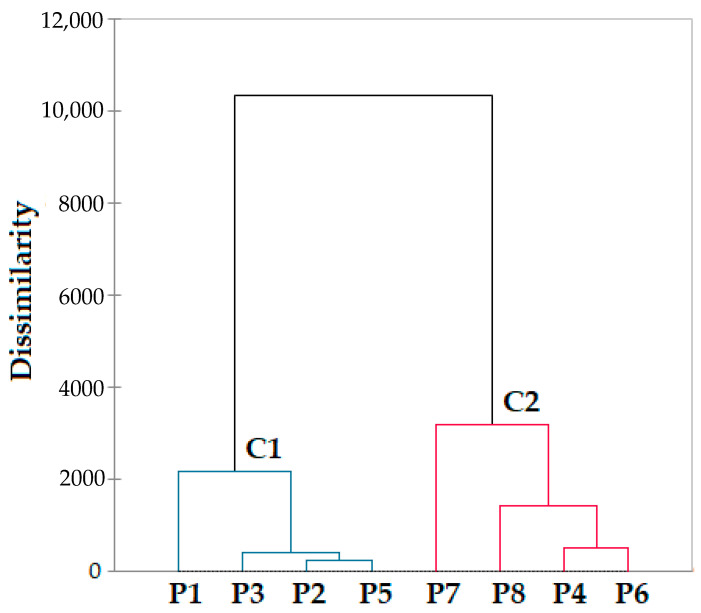
Hierarchical clustering (dendrogram) of different paprika varieties.

Hierarchical clustering (dendrogram), AHC of the paprika varieties presented in Figure 5, was conducted to find the inter-connectivity and closeness of the studied paprika samples and individual volatile organic compounds. The dendrogram cluster is divided into two groups, the first containing P1, P2, P3 and P5 and the second which contains P4, P6, P7 and P8. Paprika species from Romania and Turkey have similar chemical compositions, whereas samples from China, India, Morocco and Hungary can be differentiated. Pearson correlation regarding fatty acids confirms a very strong correlation between P1, P5, P2 and P3 and P7, P8, P4 and P6. The cluster analysis shows the differences between samples separated in each cluster based on the different content of fatty acids and volatile compounds.

**Figure 6:**
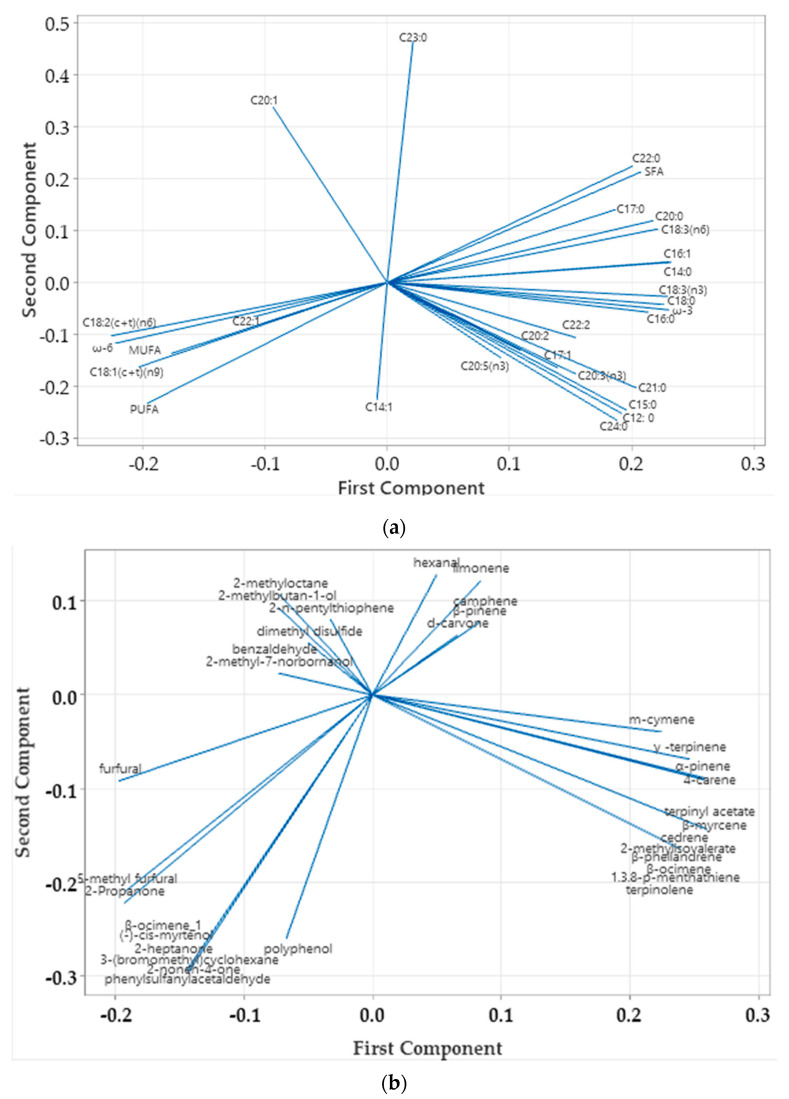
Principal component analysis loading plot of PC1 and PC2 for fatty acids (**a**) and volatiles and phenols (**b**) of different paprika varieties.

Figure 6 presents the principal component analysis regarding fatty acid, volatile compound and polyphenol correlation. The PCA is an unsupervised method to visualize the difference/similarity among sample profiles and detect significant variables contributing to these discrepancies among the eight paprika types. The data for PCA were evaluated for each class of fatty acids, volatile compounds and polyphenols as a preliminary test. Very strong positive correlations were obtained for C18:3(n6) with C16:1 (0.97), C18:0 (0.95) and C14:0 (0.92), and it was negatively correlated with C18:2(c + t)(n6) (−0.98). In addition, a strong positive correlation was obtained for C18:3(n3) with C18:0 (0.93), C16:1 (0.96) and C14:0 (0.95), and it was strongly negatively correlated with C18:2(c + t)(n6) (−0.95). A moderate correlation was obtained for C23:0 with C20:1 (0.71) (Figure 6a). Linoleic acid, C18:2, was positively correlated with the 18:1 isomer (0.95). Finally, C14:1 was positioned alone and was not related to any other components in the PCA.

In the case of volatile compounds, four clusters can be observed. A very strong positive correlation was obtained for d-carvone with camphene and β-pinene (0.99). Four clusters of volatile compounds were identified (Figure 6b). The presence of aldehydes, alcohols, esters, hydrocarbons and ketones contributes to the flavor of paprika varieties originating from different countries.

## 4. Conclusions

This study conducted a comparative analysis of volatile compounds, fatty acids, cellulose, hemicellulose, lignin and total polyphenols and evaluated the thermal behavior among paprika samples from different countries. The thermal analysis of paprika samples was in agreement with the moisture evaporation of adsorbed water and solvent (at 64–73 °C and a mass loss of 3.6–4.6%), decomposition of volatile compounds (206–214 °C and mass loss of 29.6–33.7%), decomposition of fatty acids and proteins (at 324–344 °C and mass loss of 22.1–24.5%) and degradation of cellulose, hemicellulose and lignin (at 493–489 °C, 552–560 °C and 590–606 °C and mass loss of 33.7–41.5%). In total, 32 volatile compounds divided into seven chemical classes were identified in the samples, including 16 hydrocarbons, 5 aldehydes, 4 ketones, 3 alcohols, 2 esters, 1 sulfur compound and 1 heterocyclic compound. The predominant aromas in paprika samples were citrus (29%, limonene) and woody (28%), and other aromas identified in smaller quantities were green (18%), fruity (11%), gasoline (10%) and floral (4%). The limonene was the most abundant in the paprika samples, although its amount varied between samples as follows: P7 (100%) > P8 (42.8%) > P6 (41.6%) > P3 (30.2%). The highest PUFA (ω-6) quantities were found in hot pepper from Turkey, India, China and Hungary. Linoleic acid (C18:2(c + t)) is the major PUFA fatty acid. The highest SFA content was found in golden pepper. The highest content of polyphenols was measured in P3 (10.9 g GA/kg), and the lowest content was measured in P8 (5.11 g gallic acid/kg). This could be influenced by pepper variety and also be dependent on the time of harvesting and processing. The obtained results will help create new research perspectives regarding the use of paprika powders in the food, cosmetic and pharmaceutical industries, thus creating new natural ingredients and bioactive compounds.

## Figures and Tables

**Table 1:** Retention time, volatile organic compounds, group, odor type and content (%) identified using HS-SPME GC-MS for P1–P8 paprika samples expressed as averages ± standard deviation (*n* = 3).

R_t_ (min)	Volatile Compounds	Chemical Group	Odor Types	P1	P2	P3	P4	P5	P6	P7	P8
5.1	2-methylbutan-1-ol	alcohols	green	17.2 ± 1.9	9.5 ± 1.0	<0.02	<0.02	<0.02	<0.02	<0.02	<0.02
5.2	dimethyl disulfide	sulfur comp	green	<0.03	<0.03	<0.03	<0.03	17.2 ± 1.8	<0.03	<0.03	<0.03
6.5	2-methylisovalerate	esters	fruity	<0.05	<0.05	<0.05	1.3 ± 0.1	<0.05	<0.05	<0.05	<0.05
7.4	hexanal	aldehydes	green	<0.02	<0.02	<0.02	2.7 ± 0.3	<0.02	5.3 ± 0.7	<0.02	18.6 ± 2.2
8.9	furfural	aldehydes	woody	28.1 ± 3.1	52.4 ± 5.9	44.3 ± 4.6	<0.03	53.5 ± 6.1	<0.03	<0.03	<0.03
10.2	2-methyloctane	hydrocarbons	gasoline	54.7 ± 6.4	17.2 ± 1.8	<0.06	<0.06	11.1 ± 1.2	<0.06	<0.06	<0.06
11.2	2-heptanone	ketones	fruity	<0.04	<0.04	7.1 ± 0.8	<0.04	<0.04	<0.04	<0.04	<0.04
12.0	2-Propanone	ketones	fruity	<0.03	4.4 ± 0.5	6.7 ± 0.7	<0.03	3.6 ± 0.4	<0.03	<0.03	<0.03
12.6	4-carene	hydrocarbons	fruity	<0.05	<0.05	<0.05	2.1 ± 0.2	<0.05	1.6 ± 0.2	<0.05	<0.05
12.8	α-pinene	hydrocarbons	woody	<0.02	<0.02	<0.02	6.3 ± 0.7	<0.02	4.9 ± 0.5	<0.02	<0.02
13.0	3-(bromomethyl)cyclohexane	hydrocarbons	woody	<0.03	<0.03	2.5 ± 0.3	<0.03	<0.03	<0.03	<0.03	<0.03
13.4	camphene	hydrocarbons	green	<0.02	<0.02	<0.02	<0.02	<0.02	2.5 ± 0.3	<0.02	<0.02
13.8	benzaldehyde	aldehydes	woody	<0.02	3.9 ± 0.4	<0.02	<0.02	<0.02	<0.02	<0.02	<0.02
14.0	5-methyl furfural	aldehydes	woody	<0.03	5.2 ± 0.5	6.9 ± 0.7	<0.03	3.9 ± 0.4	<0.03	<0.03	<0.03
14.1	2-methyl-7-norbornanol	alcohols	fruity	<0.05	3.3 ± 0.4	<0.05	<0.05	<0.05	<0.05	<0.05	<0.05
14.3	β-phellandrene	hydrocarbons	fruity	<0.03	<0.03	<0.03	11.1 ± 1.2	<0.03	<0.03	<0.03	<0.03
14.4	β-ocimene	hydrocarbons	fruity	<0.02	<0.02	<0.02	10.3 ± 1.1	<0.02	<0.02	<0.02	<0.02
14.5	β-pinene	hydrocarbons	woody	<0.04	<0.04	<0.04	<0.04	<0.04	4.2 ± 0.5	<0.04	<0.04
15.0	(-)-cis-myrtenol	alcohols	green	<0.02	<0.02	2.8 ± 0.3	<0.02	<0.02	<0.02	<0.02	<0.02
15.0	β-myrcene	hydrocarbons	woody	<0.02	<0.02	<0.02	3.7 ± 0.4	<0.02	<0.02	<0.02	<0.02
15.5	1.3.8-p-menthathiene	hydrocarbons	woody	<0.04	<0.04	<0.04	2.5 ± 0.3	<0.04	<0.04	<0.04	<0.04
15.7	terpinyl acetate	esters	green	<0.02	<0.02	<0.02	12.1 ± 1.2	<0.02	3.2 ± 0.3	<0.02	<0.02
16.2	m-cymene	hydrocarbons	fruity	<0.05	<0.05	<0.05	8.1 ± 0.8	<0.05	10.8 ± 1.1	<0.05	<0.05
16.3	limonene	hydrocarbons	citrus	<0.04	4.1 ± 0.4	5.4 ± 0.5	30.2 ± 3.1	7.5 ± 0.8	41.7 ± 4.3	100 ± 9.9	42.8 ± 4.3
17.0	β-ocimene	hydrocarbons	fruity	<0.02	<0.02	5.3 ± 0.6	<0.02	<0.02	<0.02	<0.02	<0.02
17.4	γ -terpinene	hydrocarbons	fruity	<0.03	<0.03	<0.03	4.3 ± 0.4	<0.03	4.2 ± 0.4	<0.03	<0.03
18.3	terpinolene	hydrocarbons	floral	<0.02	<0.02	<0.02	1.5 ± 0.1	<0.02	<0.02	<0.02	<0.02
19.6	2-nonen-4-one	ketones	fruity	<0.03	<0.03	5.8 ± 0.6	<0.03	<0.03	<0.03	<0.03	<0.03
20.6	2-n-pentylthiophene	heterocyclic	green	<0.03	<0.03	6.4 ± 0.7	<0.03	<0.03	<0.03	<0.03	38.6 ± 3.9
22.1	phenylsulfanylacetaldehyde	aldehydes	green	<0.01	<0.01	5.1 ± 0.5	<0.01	<0.01	<0.01	<0.01	<0.01
23.2	d-carvone	ketones	floral	<0.02	<0.02	1.7 ± 0.2	<0.02	3.2 ± 0.3	21.6 ± 2.2	<0.02	<0.02
29.7	cedrene	hydrocarbons	woody	<0.03	<0.03	<0.03	3.8 ± 0.4	<0.03	<0.03	<0.03	<0.03

< Below limit of quantification (LQ).

**Table 2:** Profile of fatty acids found in paprika oils; data are expressed in % (*w*/*w*), expressed as averages ± standard deviation (*n* = 3).

Acid Type	P1	P2	P3	P4	P5	P6	P7	P8
**C12:0**	2.49 ± 0.1	3.13 ± 0.12	<0.032	0.54 ± 0.01	3.56 ± 0.12	0.91 ± 0.01	1.32 ± 0.01	0.32 ± 0.01
**C14:0**	3.61 ± 0.1	3.76 ± 0.11	3.16 ± 0.15	0.96 ± 0.01	4.70 ± 0.15	1.31 ± 0.01	1.76 ± 0.01	0.79 ± 0.01
**C14:1**	1.40 ± 0.08	1.69 ± 0.07	<0.036	<0.036	<0.036	0.64 ± 0.01	0.91 ± 0.02	2.01 ± 0.05
**C15:0**	1.56 ± 0.01	1.52 ± 0.02	<0.014	0.24 ± 0.01	1.84 ± 0.02	0.41 ± 0.01	0.64 ± 0.02	0.23 ± 0.01
**C16:0**	16.0 ± 1.1	14.6 ± 1.0	13.0 ± 1.1	13.0 ± 1.0	15.5 ± 1.2	10.6 ± 0.8	12.2 ± 1.0	11.2 ± 1.1
**C16:1**	1.93 ± 0.08	2.11 ± 0.08	1.65 ± 0.05	0.63 ± 0.01	2.13 ± 0.08	0.69 ± 0.02	0.93 ± 0.03	0.46 ± 0.01
**C17:0**	2.85 ± 0.1	1.68 ± 0.1	2.42 ± 0.08	0.31 ± 0.01	2.49 ± 0.07	1.64 ± 0.01	1.24 ± 0.03	0.65 ± 0.01
**C17:1**	1.66 ± 0.04	1.77 ± 0.05	<0.023	0.23 ± 0.01	<0.023	<0.023	<0.023	0.15 ± 0.01
**C18:0**	7.57 ± 0.2	7.82 ± 0.41	5.29 ± 0.21	3.14 ± 0.12	6.64 ± 1.3	3.40 ± 0.01	4.17 ± 0.1	3.33 ± 0.13
**C18:1(c + t)** **(n9)**	5.12 ± 0.1	6.85 ± 0.12	5.02 ± 0.31	10.4 ± 1.0	5.60 ± 1.3	14.0 ± 1.1	9.61 ± 0.2	11.3 ± 1.2
**C18:2(c + t)(n6)**	21.8 ± 1.8	20.3 ± 1.8	24.3 ± 1.5	64.8 ± 2.3	20.7 ± 1.8	61.4 ± 2.5	56.8 ± 2.3	61.9 ± 4.1
**C18:3(n6)**	4.30 ± 0.2	4.22 ± 0.13	3.75 ± 0.18	0.53 ± 0.02	3.44 ± 0.05	1.03 ± 0.02	1.43 ± 0.08	0.36 ± 0.01
**C18:3(n3)**	19.6 ± 1.2	16.68 ± 1.3	10.03 ± 0.9	1.85 ± 0.012	17.78 ± 1.1	1.80 ± 0.04	4.17 ± 0.12	2.33 ± 0.1
**C20:0**	2.82 ± 0.1	4.27 ± 0.21	3.59 ± 0.2	0.74 ± 0.02	3.42 ± 0.02	0.80 ± 0.01	1.28 ± 0.08	0.29 ± 0.01
**C20:1**	<0.0052	<0.0052	3.06 ± 0.2	0.34 ± 0.01	<0.0052	0.53 ± 0.02	0.71 ± 0.02	2.62 ± 0.1
**C20:2**	1.56 ± 0.1	<0.0096	<0.0096	0.26 ± 0.01	2.56 ± 0.05	<0.0096	1.71 ± 0.01	0.19 ± 0.01
**C21:0**	2.21 ± 0.08	3.04 ± 0.10	<0.0086	0.40 ± 0.01	3.18 ± 0.01	<0.0086	<0.0086	0.29 ± 0.01
**C20:3(n3)**	<0.0061	2.22 ± 0.11	<0.0061	0.21 ± 0.01	1.72 ± 0.01	<0.0061	<0.0061	0.13 ± 0.01
**C20:5(n3)**	<0.0058	1.46 ± 0.08	<0.0058	<0.0058	<0.0058	<0.0058	<0.0058	0.13 ± 0.01
**C22:1**	<0.0063	<0.0063	<0.0063	<0.0063	<0.0063	<0.0063	<0.0063	0.13 ± 0.01
**C22:0**	1.15 ± 0.02	1.42 ± 0.07	1.77 ± 0.08	0.33 ± 0.01	1.46 ± 0.07	0.47 ± 0.01	0.62 ± 0.01	0.26 ± 0.01
**C22:2**	1.30 ± 0.01	<0.0091	<0.0091	<0.0091	1.62 ± 0.05	<0.0091	<0.0091	0.13 ± 0.01
**C23:0**	<0.056	<0.056	21.62 ± 1.8	0.77 ± 0.01	<0.056	<0.056	<0.056	<0.056
**C24:0**	1.08 ± 0.08	1.42 ± 0.07	<0.050	0.32 ± 0.01	1.62 ± 0.1	0.43 ± 0.02	0.50 ± 0.01	0.23 ± 0.01
**SFA**	41.4 ± 3.1	42.7 ± 2.1	50.8 ± 4.1	20.7 ± 1.6	44.4 ± 2.6	19.9 ± 1.1	23.8 ± 2.0	18.1 ± 1.2
**MUFA**	10.1 ± 1.1	12.4 ± 1.0	9.7 ± 0.5	11.6 ± 1.0	7.7 ± 0.2	15.9 ± 1.4	12.2 ± 1.1	16.7 ± 1.3
**PUFA**	48.5 ± 3.1	44.9 ± 3.5	38.0 ± 2.3	67.6 ± 4.2	47.8 ± 3.1	64.2 ± 2.6	64.1 ± 6.0	65.2 ± 4.6
**ω-6**	29.0 ± 1.8	24.5 ± 1.8	28.0 ± 1.5	65.6 ± 4.3	28.3 ± 1.9	62.4 ± 5.2	59.9 ± 4.2	62.6 ± 4.2
**ω-3**	19.6 ± 1.1	20.4 ± 1.9	10.0 ± 0.9	2.1 ± 0.1	19.5 ± 1.2	1.80 ± 0.08	4.17 ± 0.3	2.59 ± 0.1
**ω-6/ω-3**	1.5 ± 0.08	1.2 ± 0.07	2.8 ± 0.1	31.8 ± 2.6	1.5 ± 0.1	34.6 ± 2.1	14.4 ± 1.2	24.2 ± 2.1

< Below limit of quantification (LQ).

**Table 3:** Total polyphenol contents in the studied paprika samples expressed as averages ± standard deviation (*n* = 3).

Paprika	P1	P2	P3	P4	P5	P6	P7	P8
Polyphenol g GA kg^−1^	8.35 ± 0.79	9.44 ± 0.96	10.9 ± 1.1	8.18 ± 0.82	5.61 ± 0.61	7.81 ± 0.81	7.44 ± 0.73	5.11 ± 0.52

## Data Availability

The data presented in this study are available on request from the corresponding author.
